# Honey as an Ecological Reservoir of Antibacterial Compounds Produced by Antagonistic Microbial Interactions in Plant Nectars, Honey and Honey Bee

**DOI:** 10.3390/antibiotics10050551

**Published:** 2021-05-09

**Authors:** Katrina Brudzynski

**Affiliations:** 1 Department of Drug Discovery, Bee-Biomedicals Inc., St. Catharines, ON L2T 3T4, Canada; beebio@sympatico.ca; 2 Formerly Department of Biological Sciences, Brock University, St. Catharines, ON L2T 3T4, Canada

**Keywords:** honey, microbiota, antimicrobial compounds, bacteriocins, surfactants, siderophores, mode of action, spectrum of activity, pathogenesis-related proteins, bee antimicrobial peptides

## Abstract

The fundamental feature of “active honeys” is the presence and concentration of antibacterial compounds. Currently identified compounds and factors have been described in several review papers without broader interpretation or links to the processes for their formation. In this review, we indicate that the dynamic, antagonistic/competitive microbe–microbe and microbe–host interactions are the main source of antibacterial compounds in honey. The microbial colonization of nectar, bees and honey is at the center of these interactions that in consequence produce a range of defence molecules in each of these niches. The products of the microbial interference and exploitive competitions include antimicrobial peptides, antibiotics, surfactants, inhibitors of biofilm formation and quorum sensing. Their accumulation in honey by horizontal transfer might explain honey broad-spectrum, pleiotropic, antibacterial activity. We conclude that honey is an ecological reservoir of antibacterial compounds produced by antagonistic microbial interactions in plant nectars, honey and honey bee. Thus, refocusing research on secondary metabolites resulting from these microbial interactions might lead to discovery of new antibacterial compounds in honey that are target-specific, i.e., acting on specific cellular components or inhibiting the essential cellular function.

## 1. Introduction

Honey possesses various antimicrobial compounds that kill or supress growth and proliferation of a broad spectrum of microorganisms including multi-drug resistant pathogens [1,2,3]. Dating from 1992, with the fundamental study of honey antimicrobial activity by Molan [3], there has been an avalanche of papers concerning activity of honeys from different geographical and botanical origins, honey chemical composition, the presumptive compounds responsible for its activity and honey therapeutic potential. This knowledge has been summarized in the most recent reviews [1,2]. However, in order to develop honey as an antibacterial biological product, novel active compounds of specific mechanism of action have to be identified. 

Most of honey antibacterial compounds identified so far target bacterial cells in a non-specific manner, including the main bactericidal compounds, hydrogen peroxide (H_2_O_2_) and methylglyoxal (MGO). The concentrations of these two compounds strongly correlate with antibacterial activity of honey with the minimum inhibitory concentration/minimum bactericidal concentration (MIC/MBC) in the µg/ml range [4,5]. The lethal effect of honey H_2_O_2_ results from the accumulation of the oxidative damages to the structure and conformation of proteins, enzymes, unsaturated fatty acids of bacterial cell membranes and the production of reactive oxygen species (ROS) that causes DNA strand breaks and DNA degradation [6,7,8,9]. On the other hand, bactericidal effect of MGO, a dicarbonyl compound responsible for antibacterial activity of Manuka honey, results from its ability to irreversibly glycate and crosslink macromolecules, proteins and DNA, respectively, leading to loss of their functions [5,10]. Antimicrobial activity of honey has been also attributed to the acquisition of secondary metabolites originated from plants such as polyphenols, flavonoids and volatile compounds [11,12]. These large and chemically diverse groups have evolved as an innate plant defence system against microbial infections and also other stressors [13,14,15]. The antimicrobial activity of plant secondary metabolites inherited from nectars becomes a significant contributor to honey antibacterial activity. However, like H_2_O_2_ and MGO, plant secondary metabolites exert non-specific, pleiotropic action against bacterial cells. Their weak antimicrobial potencies are reflected by the MIC values ranging from micro- to millimolar levels [16].

It could be argued that due to the non-selective mechanism of action of the principal antimicrobial compounds, honey displays an indiscriminate broad spectrum of antibacterial activity against bacteria, including multi-drug-resistant ones [17,18,19]. However, recent evaluation of phenotypic changes in bacterial cells treated with honey might suggest target-specific effects, for which the non-specific action of H_2_O_2_ or MGO could not be accounted for [20,21,22,23,24]. The observed effects resembled the action of β-lactams [23], antimicrobial peptides [25] or inhibitors of proton motive force and chemiosmosis [26]. These data indicate that additional sources of antimicrobial compounds of honey might exist to explain the observed changes in bacterial phenotypes. In order to move forward beyond current knowledge of honey antimicrobial activity toward elucidation of a specific mode of honey action, we propose in this review a new perspective on the origin of antimicrobial compounds in honey.

There is a mounting evidence implicating microbial ecosystem of the nectar-honey-honey bee axis involved in the production of a range of antimicrobial agents. These agents are used as weaponry in competitive interspecies interactions to effectively kill competing microorganisms in the fight for nutrients and space in each of these niches (nectar, honey and honey bee). The microbial ecosystem includes both bacteria and fungi. From a simplified, mechanistic point of view, antimicrobial compounds produced by microbiota of nectar and bee that are released to their growing media can ultimately accumulate in honey by horizontal transfer. Recent work from several laboratories has documented the impact of the microbial compounds on antimicrobial activity of honey.

With this in mind, the review is aimed to provide support for the hypothesis that the antimicrobial compounds of microbial origin comprise the novel source of honey active ingredients. Among secondary metabolites produced by these microorganisms are antimicrobial peptides, bacteriocins, surfactants, siderophores, proteolytic and cell wall-degrading enzymes. By targeting the crucial cellular structures and through different modes of actions (pore- formation, membrane solubilisation or iron- sequestration, to name a few), they affect structural integrity and function of competing microorganisms by preventing surface attachment and biofilm formation, disrupting quorum sensing thereby affecting gene expression. The range and levels of secreted antimicrobial compounds depends on the composition of microbes involved in the antagonistic interactions. Therefore, this review is organized into three main sections: Part A—describing honey microbiome; Part B—presenting antimicrobial compounds produced by honey microbiota; and Part C—providing a brief overview of antagonistic microbe–host interactions. Available literature data allow reviewing only the antimicrobial compounds of microbes that have been currently identified in honey or honey bees. Despite that limitation, the presented evidence has shown that products of microbial competitions in nectar, honey and honey bees shape honey antibacterial activity. These findings may be the starting point for more vigorous investigations into detection of antimicrobial compounds of microbial origin in honey and their relevance to the mechanisms of honey antimicrobial activity.

## 2. Antagonistic Interspecies Interactions as a Source of Antimicrobial Compounds

Microbial colonization of nectar, honey and bees is at the center of antagonistic interspecies interactions. The niche overlap by bacteria and fungi in environments often results in interspecies competitions to limit the growth of other contestants and to increase their own chances of survival [27,28]. Phylogenetically related species (for example, *Bacillus* and *Lactobacillus* spp.) that reside under similar conditions in enclosed environments, such as in unripen honey in the honeycomb, tend to interact vigorously with each other in a competitive manner to increase their access to limited space and carbohydrate resources. Consistently with the interference and exploitive competitions, they synthesize and secrete molecules directed to damage key cellular structures and functions of competing microorganisms. In particular, compounds that target cell wall integrity, cell wall synthesis, energy production, iron sequestration or ion efflux/influx significantly impact cell viability [27,28]. On the other hand, the functional capacity of competing strains to withstand antagonistic actions depends on increased proliferation to gain a space advantage and to reach high cell density. This is achieved by the secretion of signalling molecules, autoinducers, that increase cell proliferation to obtain a critical cell concentration, a quorum. Quorum sensing (QS) is a vital regulatory mechanism that upregulate gene expression of antimicrobials such as bacteriocins, iron scavengers and biofilm formers thereby allowing bacteria to withstand the attack and exploit the niche. Moreover, QS-dependent biofilm formation provides protection to the members of the population and also serves as a store of nutrients [29]. QS is also responsible for the activation, synthesis and secretion of virulence factors that underlie bacterial expansion and pathogenicity [29].

**Figure 1:**
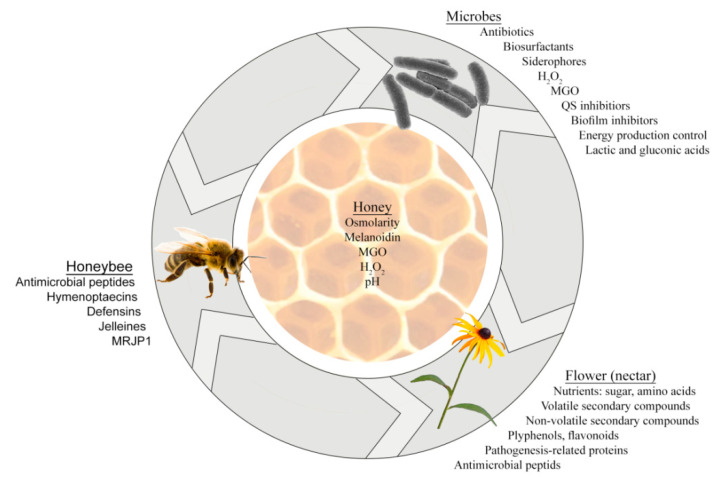
Three-way interactions between microbes, plants and honey bees during which the secondary metabolites are produced and contribute to honey antimicrobial activity.

Together, microbes of the nectar–honey–honey bee axis produce multiple active secondary metabolites, having different modes of action [30], and displaying pleiotropic actions on growth, metabolism, gene expression, colony formation, biofilm production that ultimately affect cell viability (Figure 1). By accumulation in honey, these compounds add to its antibacterial activity.

## 3. Discovery of Antibacterial Activity of Honey-Associated Microbiota 

The presence of microbial contaminants in honey has been known for decades and recognized as a safety hazard that could lead to foodborne diseases [31,32,33]. However, current research shows that some naturally occurring microbes in honey, including genera *Lactobacillus* and *Bifidobacterium*, *Bacillus* or yeast (*Saccharomyces cerevisiae*), are useful in prevention of food spoilage due to the production of antimicrobial compounds.

Pioneering studies by Worobo group have shown that microbial contaminants of raw, unsterilized honeys produced antimicrobial compounds able to inhibit a range of food spoilage microorganisms and human pathogens, among them, *Aspergillus niger*, *Penicillium expansum*, *Lactobacillus acidophilus*, *Pseudomonas fluorescens*, *Bacillus cereus*, *Escherichia coli O157:H7*, *Listeria monocytogenes*, *Salmonella enterica Ser. Typhimurium*, *and Staphylococcus aureus* [34]. In these studies, more than 90% of bacterial strains of honey displayed in vitro antimicrobial activity against reference bacteria, as well as antifungal activity against mold, *Byssochlamys fulva* H25 [35]. These findings were a significant step forward in recognizing that the presence of bacterial strains exhibiting antimicrobial activity in honeys was a wide-spread phenomenon and had to be taken for account when considering the mechanism of honey antibacterial activity [36].

**Part A**

## 4. Honey Microbiome

**Figure 2:**
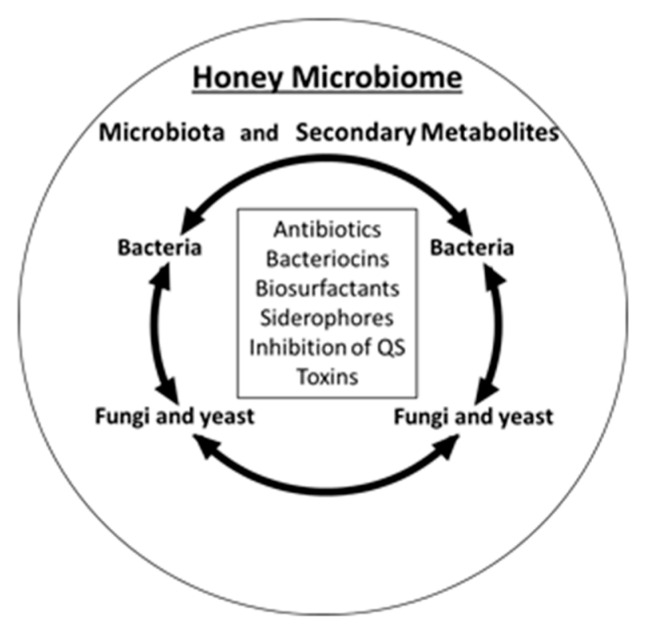
Honey microbiome presented as a combination of the microbial occupants of honey and the secondary metabolites they produce as a result of antagonistic, interspecies interactions and secrete to growth medium (honey) (figure adapted from [37]).

Microbial colonization of nectar, honey and honey bee is the main factor shaping the composition of honey microbiota. Who is there is important for metabolites they produce through exploitive and interference competition. In turn, the pool of antimicrobial compounds secreted to honey shapes both, honey microbiota by eliminating the competing microorganisms, and honey antimicrobial activity. These relationships underlie the formation of honey microbiome (Figure 2). Honey microbiome combines honey/nectar microbiota and their metabolites. Among honey core microbiota are two dominant orders of bacteria, Lactobacillales and Bacillales (the genera *Bacillus* and *Paenibacillus*) and dominant species of fungi and yeasts.

## 5. The Core Bacteria of Honey

The primary sources of microbial contamination of honey are air, water and pollinating environments (nectar and pollen) but the primary route of contamination are bees whose foraging activities spread microbes among flowers [38,39] and then bring contaminated food, nectar and pollen, to the hives. Diversity and composition of nectar and pollen microbiota is reduced during conversion of nectar to honey. Honey ripening and the changes in physicochemical conditions, eliminates the most of transient microbial contaminates [40,41]. The gradual water evaporation, acidification and increasing sugar concentration serve as selecting factors for osmotolerant, xerotorelant and acidotolerant microorganisms, shaping the composition of core honey microbiota. The metagenomics analysis using culture-dependent and independent methods have revealed that the composition of core microbiome of honey shows an extensive overlap with microbiomes of nectar, pollen and the honey bee stomach, crop. The core phylotypes includes Actinobacteria, Firmicutes, Proteobacteria (Alpha- and Gammaproteobacteria) (Table 1). However, the families of Bacillaceae, Lactobacillaceae are the most prevalent in honey, followed by Enterobacteraceae, Acetobacteraceae, Microbacteriaceae and Bifidobacteriaceae [42,43,44,45]. Although the composition of honey microbiota might differ with plant botanical and geographical origins [33,38,39], the keystone species in the core microbiota of honey are *Bacilli* and *Lactobacilli*, and their products of antagonistic interspecies interactions are functionally relevant to honey activities.

### 5.1. The Composition Lactic Acid Bacteria in Honey

Bacteria of the genus *Lactobacillus* and *Bacillus* are specifically abundant in environments rich in carbohydrates. Among them, fructose-rich niches such as honey and other beehive products are frequently colonized by lactic acid bacteria (LAB) and fructophilic lactic acid bacteria (FLAB) [46,47,48]. Research of Olofsson group succeeded in identification of several LAB and FLAB in honey stomach (crop) and in honey [48,49]. The crop serves as a means to transport of collected nectars to the hive. As a part of the honey bee foregut, the crop is used as a site of food storage and an initial site for the digestion of carbohydrates by its resident LAB microbiota. Thus, the main source of LAB in honey is pollination environment (nectar and pollen) and the bee foregut [42,43,45,49,50].

The most frequently isolated species of FLAB in nectars, honey and honeydew include *L. kunkeei*, *L. apinorum*, *L. mellis*, *F. fructosus*, *L. apis*, *L. mellifer*, *L. melliventris*, *L. johnsonii*, *L. plantarum*, *L. brevis*, *L. kimbladii*, *L. helsingborgensis* and *L. kullabergensis* [46,47,48,49,50,51] (Table 2).

The listed members of LAB have a significant influence on antimicrobial activity of beehive products.

Several LAB and FLAB isolated from pollen, honey, bee bread and crop displayed antimicrobial activities against bee pathogens, foodborne and multi-drug-resistant human pathogens [47]. Isolates of *L. johnsonii*, *L. plantarum*, *L. brevis*, *L. apis* inhibited growth of *Melissococcus plutonius* and *Paenibacillus larvae*, the causes of European and American foulbrood diseases, respectively [46,52]. *L. acidophilus* strains isolated from Malaysian honey were able to inhibit multiple antibiotic resistant *Staphylococcus aureus*, *Staphylococcus epidermis* and *Bacillus subtilis* [53,54], while anti-biofilm activity of *L. kunkeei* effectively block biofilm development and infection caused by *P.* *aeruginosa* in vivo [55].

LAB are well-recognized as the producers of the most active anti-fungal compounds against filamentous fungi, *Aspergillus* and *Penicillium* spp. and yeasts, *Saccharomyces*, *Candida*, *Kluyveromyces*, *Zygosaccharomyces* and *Pichia*, spp. Lactobacilli isolated from beebread such as *L. kunkeei*, *F. fructosus* and *F. tropaeoli* have shown a strong antagonistic action against *Zygosaccharomyces rouxii* [53]. *Zygosaccharomyces rouxii* and *Zygosaccharomyces bailii* are osmotolerant and stress-resistant food- spoilage yeasts that can grow and ferment honey if the moisture content of honey increases over 18% [56]. Thus, the anti-fungal activity of LAB could protect honey against spoilage [57]. Importantly, LAB have ability to remove or inactivate mycotoxins produced by *Aspergillus*, *Fusarium*, and *Penicillium.* Research showed that the potent mycotoxin, Aflatoxin B1, of *Aspergillus* can be inactivated and removed by a spontaneous binding/adherence to the cell wall of viable or non-viable *Lactobacillus* spp. such as *L*. *rhamnosus* [58]. The second mechanism of removal mycotoxins by microorganisms in natural way is via its metabolic conversion to harmless, non-toxic derivatives. This mechanism operates efficiently in Bacillaceae (see below). Anti-fungal activity of LAB and their participation in mycotoxins inactivation might play role in honey preservation and safety.

### 5.2. The Composition of the Family Bacillaceae in Honey

*Bacillales* are another, dominant order of Firmicutes colonizing nectar and honey. A significant percentage of honey microbiota, ranging from 60% to 90% of all bacteria in honey, is composed of *Bacillales* including genera *Bacillus* and *Paenibacillus* [55,56,57,58,59]. 16S rRNA gene sequenceing and MALDI-TOF revealed that the bulk of the *Bacillus* isolates belonged to three phylogenetic clusters: (1) *Bacillus subtilis* group comprising of *B. subtilis*, *B.* *methylotrophicus*, *B. atrophaeus*, *B. licheniformis* and *B. amyloliquefaciens*, (2) *B. cereus* group including, *B. cereus*, *B. thuringiensis*, *B. mycoides*, *B. pseudomycoides* and *B. weihenstephanensis* and (3) *B. pumilis* represented by *B. pumilis*, *B. safensis*, *B. altitudinis* [59,60,61,62] (Table 3).

All these *Bacillus* strains produce gene-coded and non-ribosomally synthesized antimicrobial peptides, as discussed below [63,64]. Together with LAB, *Bacillus* spp. comprise an efficient factory that supply honey with a broad range of antimicrobial compounds. Antagonistic interspecies interactions are key to their production.

The unusually high antibacterial activity of Polish honeys, specifically against *Staphylococcus aureus*, has been linked to the significant antimicrobial activity of *Bacillus* spp. [65]. Over hundreds of bacterial strains have been isolated from these honeys and screened for their growth inhibitory activities. Most of bacterial strains demonstrated broad spectrum of antimicrobial activity, with bacteriostatic activity against several reference strains of *Staphylococcus aureus*, *Staphylococcus epidermis*, *Escherichia coli*, *Listeria monocytogenes*, *Pseudomonas aeruginosa* and *Candida albicans* [60,65].

### 5.3. Fungal Composition of Nectar and Honey

Yeast and fungi are the most abundant group of microbes contaminating nectars and honey [31,33,66]. Their diversity and richness are reduced by physicochemical conditions of nectar and honey, that is, low pH, acidity and high sugar concentration, and by antagonistic, competitive interactions between microbes. Surviving yeast and fungi are osmotolerant, xerotolerant and acidotolerant [67,68]. Phylogenetic analyses of nectars indicated that the most abundant group of fungal contaminants were ascomycetous yeasts (genera *Candida*, *Eremascus*, *Metschnikowia*, *Bettsia*, *Monascus*, *Oidiodendron*, *Pichia*, *Saccharomyces*, *Skoua*, *Torulopsis*, *Zygosaccharomyces*) and ascomycetes filamentous fungi (genera *Aspergillus*, *Penicillium*, *Cladosporium*, *Mycelia sterilia* and *Fusarium*) with less frequent occurrence of genera *Arthrinium*, *Chaetonium*, *Daldinia or Emericella* [59,68] (Table 4). For example, the xerotolerant *Bettsia*, *Ascosphaera*, *Metschnikowia* and *Eremascus* can survive at very low water activity (up to 0.82), while the acidophilic or acidotolerant *Pichia*, *Saccharomyces* and *Zygosaccharomyces* can grow even below pH 2 [31,67] (Table 4).

Their survival strategies include (a) the formation of spore allowing them to survive the adverse conditions in the dormancy state and (b) the production of secondary metabolites with antimicrobial activities [69,70]. Among the antimicrobial compounds produced by fungi are those considered beneficial from the perspective of human health such as antibiotics [71] but also deleterious, such as killer toxins (mycotoxins) [72]. While the latter compounds have direct, lethal effects on microbial competitors, some other compounds such as siderophores or surfactants inhibits their growth by interfering with supply of iron or with the signalling in quorum sensing and the formation of protective biofilms (see below).

In summary, honey appears as a rich reservoir of microbes and can be viewed as a heterogeneous microbial ecosystem containing yeast, molds and bacteria. The composition of honey microbiota is a key factor determining the repertoire of antimicrobial compounds produced via antagonistic interactions.

**Part B**

## 6. The Overview of Antimicrobial Compounds Produced by Honey Microbiota

Implication of microbial “contaminants” in the antimicrobial activity of honey-initiated efforts to isolate and identify the compounds responsible. Due to dominance of the families of Lactobacillaceae and Bacillaceae, the antimicrobial compounds in honey were expected to be largely produced by members of these families. Lactobacillaceae and Bacillaceae are known producers of antimicrobial compounds such as bacteriocins, surfactants and siderophores. Diversity, structure, classification and modes of action of these compounds have been extensively reviewed in literature [63,64,73,74,75]. The current review is focused on antimicrobial compounds produced or predicted to be produced by the bacterial strains of LAB and *Bacillus* spp. identified in honey thus far. With the fast progress in identification of new bacterial strains and new antimicrobial compounds in honey, it is expected that the current depiction of antimicrobial compounds in honey will evolve and enrich the data presented here. 

### 6.1. LAB Bacteriocins

Bacteriocins are small, ribosomally-synthesized cationic antimicrobial peptides produced during bacterial logarithmic growth of *Lactobacillus*, *Lactococcus*, *Pediococcus* and *Leuconostoc* [76].

The structure, classification and mode of action of LAB bacteriocins have been a subject of several excellent reviews [73,74,75,76,77]. However, despite a significant representation of LAB in the bee products, so far, only one bacteriocin, kunkecin A, has been isolated from *L. kunkeei* of honey bee [78]. There are some indications that in addition to *L. kunkeei*, several other lactobacilli (*L. johnsonii*, *L. plantarum*, *L. brevis*, *L. apis*) might produce bacteriocins since they demonstrated bactericidal activity against honey bee pathogens, *Melissococcus plutonius* and *Paenibacillus larvae*, the causes of European and American foulbrood diseases, respectively [47,53].

#### 6.1.1. Kunkecin A

**Figure 3:**
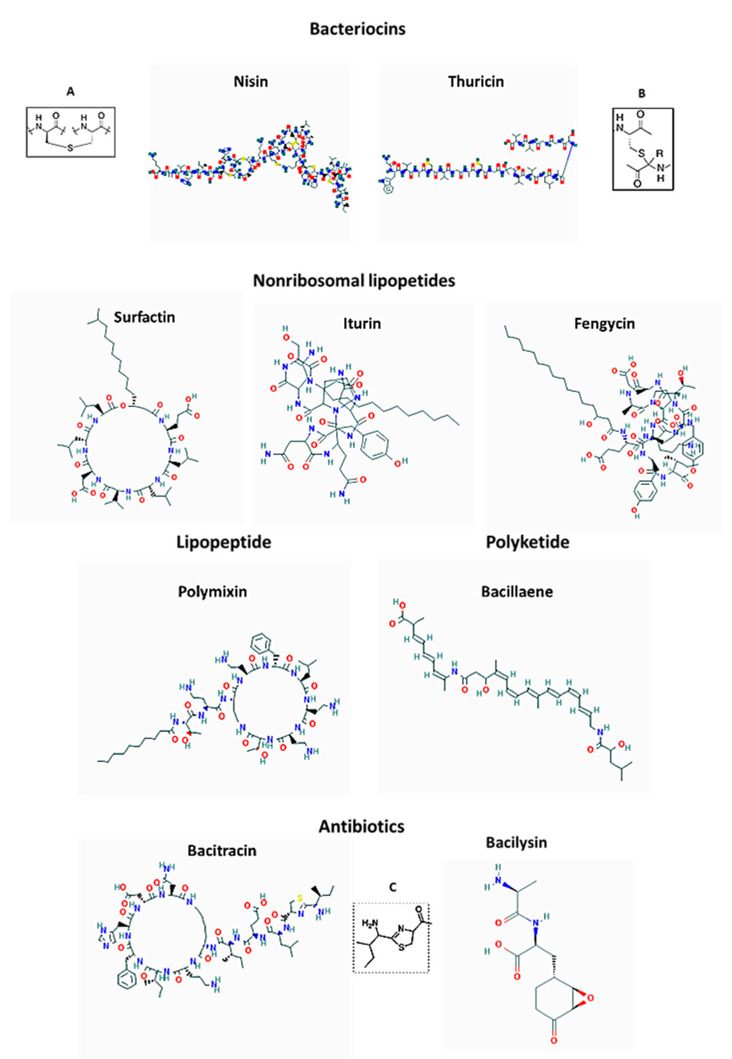
Structures of bacteriocins, antibiotics, lipopetides and polyketide of *Bacillus* spp. Inserts present; (**A**) lanthionine ring, (**B**) sactibiotic ring, (**C**) thiazoline ring.

Kunkecin A is a variant of nisin A, a lantibiotic bacteriocin produced by *Lactococcus lactis* subsp. *lactis* [78]. Both these bacteriocins belong to class I A lantibiotics and contain thioether amino acids lanthionine and methyllanthionine as the key structural signature (Figure 3). The cellular target for these bacteriocins is lipid II of the cell wall of Gram-positive bacteria. The lantionine rings at the N-terminus of these cationic peptides play a crucial role in the binding to anionic phosphate groups of lipid II of the cell wall of Gram-positive bacteria. The binding initiates the process of pore-formation [79].

#### 6.1.2. Mode of Action

Although the exact mechanism of pore-formation by lantibiotic bacteriocins is still unresolved, it is apparent that the main steps in the process include (a) the change of orientation of the peptide-lipid II complex from parallel to perpendicular with respect to the membrane, (b) the insertion of the C-terminal of the peptide into the cytoplasmic membrane and (c) the formation of transmembrane, water- filled pore [80]. In the case of nisin, the insertion into the membrane depends on a critical concentration of bacteriocin-lipid II complexes and the electrical transmembrane potential (Δ*ψ*) and transmembrane pH gradients (Δ*pH)* [62,76,81]. It is hypothesized that aggregated bacteriocins change/inverse their surface charge because of pH changes (pH gradient, Δ*pH)* or because of the difference in the membrane electric potential (Δ*ψ*) caused by the transmembrane proton motive force (PMF) and form a pore as the result of the electrostatic repulsion between individual peptides. The formation of pores causes the efflux of ions, amino acids, and ATP from cells, thereby collapsing the proton motive force, and with it the ATP synthesis and collapsing pH gradient that regulate ion exchange between cell interior and exterior. Ultimately, the pore formation inhibits the cell function while increased cell permeabilization lead to rapid cell death [62,76,81,82,83].

The second mode of action of lantibiotics includes inhibition of cell wall biosynthesis through the binding to lipid II. Lipid II is the main transporter of a peptidoglycan subunit from the cell interior to the place of cell wall synthesis. Biding of nisin to lipid II causes withdrawal of lipid II from the cytoplasm-cell wall circulation and delivery of peptidoglycan subunits to the growing peptidoglycan chain [79,83]. Consequently, the dual mechanism of action of these bacteriocins that combine the inhibition of peptidoglycan synthesis with pore formation result in a potent bactericidal activity with MICs at nanomolar concentrations [76,79,83]. Whether or not the mode of action of kunkecin A is similar to other lantibiotics remains unknown at this time.

#### 6.1.3. Spectrum of Antimicrobial Activity

Nisin has a broad spectrum of antibacterial activity against Gram-positive bacteria, including staphylococci, streptococci, bacilli, clostridia, and mycobacteria [84]. In contrast, kunkecin A seemed to specifically target *Melissococcus plutonius*, a causative pathogen of European foulbrood [47,77].

The mechanism of action of lantibiotics via lipid II binding of the cytoplasmic membrane makes Gram-positive bacteria their preferentially target. Lantibiotics show poor antibacterial activity against Gram-negative bacteria due to the presence of the outer membrane (OM) that is a penetration barrier for the peptides to access the cytoplasmic membrane. However, lantibiotic can destroy Gram-negative bacteria if the OM integrity is compromised, for example, by chelating agents, such as EDTA [76,82,84]. The OM of Gram-negative bacteria composed of polyanionic lipopolysaccharide is stabilized by divalent cations (Mg^2+^ and Ca^2+^). Chelation of the ions by EDTA is a common method to increase the OM permeability to antibiotics [85]. For example, nisin has been shown to inhibit the growth of Gram-negative bacteria more efficiently in the presence of chelating agents [86,87].

Several members of the genus *Lactobacillus* produce bacteriocins that have a rapid bactericidal activity against human pathogens (Table 5). For example, *Lactobacillus helveticus* and *Lactobacillus plantarum* isolated from bee products showed bactericidal activities against *Staphylococcus aureus* and *Acinetobacter baumannii* that has been presumably associated with the production of bacteriocins, helveticin J and plantaricin, respectively [47,52]. Other bacterial strains of lactobacilli isolated from the bee crop showed bactericidal activity against pathogens isolated from human wounds such as methicillin-resistant *S. aureus* or *P. aeruginosa*, vancomycin-resistant *Enterococcus* and *E. coli* [88,89]. The strains of *Lactobacillus acidophilus* isolated from Malayasian honey showed bactericidal activity against multi-drug resistant *S. aureus* and *S. epidermis* that was suspected to be associated with acidocin [53]. 

In summary, isolates of the *Lactobacillus* genera have been shown to produce bacteriocins exhibiting a high antibacterial activity in the nanomolar range. They showed narrow spectrum activity directed mostly to closely related strains (Table 5). Several studies indicated that lactobacilli similar to those isolated from honey and bee products displayed a rapid bactericidal activity.

### 6.2. LAB Surfactants, Modes of Anti-Biofilm Action and Spectrum of Activity

In addition to antimicrobial action of bacteriocins, LAB produce biosurfactants, a diverse group of amphipathic molecules that can modify physicochemical properties and integrity of the cell envelope by interacting with its components. The chemical nature of biosurfactants originated from different LAB strains ranges from proteinaceous compounds, glycolipidic, glycoproteins, to glycolipopeptides [94,95,96,97,98]. The key to their surfactant action is their amphipathic nature conferred by charge, hydrophobicity and degree of chemical modifications [94,95,96,97,98]. Their interactions with membranes can lead to the reduction of the surface tension, a partial membrane solubilisation or change in membrane hydrophobicity and/or cell wall charge [94]. The changes in properties of the cell envelope affect biofilm formation, specifically the ability of bacterial cells to attach to the surfaces. The anti-biofilm activity of biosurfactants can result from a direct inhibition of biofilm formation, but also from the dispersal of the mature biofilms [95,97,98]. Biofilm development is the important microbial strategy in exploitive competition for nutrients acquisition and storage, the space colonization, spread and defence against the competition or host defences. Prevention of biofilm formation and degradation of mature biofilms has a serious repercussion to the bacterial survival by the disruption of quorum sensing (QS) [99,100]. Quorum sensing (QS) system has a critical role in regulation of gene expression required for normal cell functions, colonization and pathogenicity [29,101]. In addition to controlling of gene expression of surface-associated adhesins and biofilm formers, QS system control expression of many virulence factors involved in pathogenicity, including antibiotics, autolysins associated with cell division, bacteriocins and haemolysins [100,101,102]. It also regulates motility and sporulation in *Bacillus* spp. [29,101]. Together, the disruption of QS by biosurfactants drastically reduces microbial chances for survival.

#### 6.2.1. Mode of Action

Because of the lack of detailed structural data, the mode of action of LAB biosurfactants is deduced from the final effect on biofilm formation. Moreover, the determination of the exact mode of action is also hampered by the fact that most of the studies on the effects of biosurfactants were performed on planktonic cells that show higher sensitivity to antimicrobial agents that those embedded and protected by biofilms. In general, biosurfacatant effects that have been commonly observed include (a) the reduction of attachment of competing pathogens to the surfaces of medical devices, (b) the degradation of biofilms followed by the rapid bactericidal effect and (c) biofilms’ co-aggregations [98]. For example, biosurfactants produced by *L. lactis*, *L. acidophilus*, *L. fermentum*, *L. casei*, *L. rhamnosus* displayed anti-adhesion activity [96]. This property has been used in pre-coating of biomedical instruments and implants with the LAB biosurfactants to prevent attachment and biofilm formation by pathogenic bacteria, such as *S. aureus*, *P. aeruginosa* or *C. albicans* [103]. Biofilms of pathogenic bacteria developed on catheters, cardiac pacemakers, joint prosthetics, etc., are responsible for about 50% of hospital nosocomial infections [95,99]. Some of surfactants facilitated desorption of existing biofilms [95]. For example, biosurfactant produced by *L. helveticus* MRTL91 displayed a strong antimicrobial activity against biofilms *of E. coli*, *C. albicans*, *P. aeruginosa*, *S. typhi*, *S. flexneri* but in contrast rather weak antiadhesive activity suggesting the possibility of bacteriocins involvement in the biofilm degradation and bactericidal activity [103].

Based on the collected data, the mechanism by which bacteriocins inhibit biofilm formation has been proposed to include (a) changing hydrophobicity of the cell envelope that is required for adhesion, (b) by pore formation and (c) by dispersal of mature biofilms due to lysis of sessile cells of biofilm [104].

In addition to inhibition of biofilm formation and biofilm dispersal by bacteriocins, biosurfactants isolated from *Lactobacillus plantarum* and *Pediococcus acidilacti* caused downregulation of gene expression of biofilm-related genes (*cidA*, *icaA*, *dltB*, *agrA*, *sortaseA* and *sarA*) in *S. aureus* as indicated by real-time RT-PCR and scanning electron microscopy. The involvement of biosurfactants in quenching of QS system was further extended to their role in the downregulation of the gene expression of autoinducer-2 (AI-2) involved in QS signalling [105].

Co-aggregation of biofilms is another anti-biofilm mode of action. Biofilm produced by the *Lactobacillus* genera showed the ability to co-aggregate with biofilms of pathogenic bacterium. This co-aggregation property is currently used in food industry to prevent foodborne pathogens to establish their own biofilms [106].

#### 6.2.2. Spectrum of Activity

Anti-biofilm activity of biosurfactants produced by several *Lactobacillus* spp. show broad spectrum of activity against Gram-positive, Gram-negative bacteria, multi-drug resistant clinical isolates and antifungal activity [103]. Biosurfactants inhibited the biofilm formation of *S. aureus*, *L. monocytogenes*, *E. coli* O157: H7 and *S. enterica* subs *enterica* serovar Typhimurium in dose-dependent manner [105,107,108]. The cell-bound biosurfactant of *Lactobacillus agilis* CCUG31450 showed antimicrobial activity against *S. aureus*, *S. agalactiae* and *P. aeruginosa* [98].

## 7. Antimicrobial Compounds Produced by *Bacillus* spp.

The family Bacillaceae dominates honey microbiota due to a high capacity of production of antimicrobial compounds that are directed against competitive microorganisms. Members of the genera *Bacillus* and *Paenibacillus* produce one or more antimicrobial compounds belonging to gene-coded bacteriocins, or/and non-ribosomally synthetized lipopeptides, (sufactants) and siderophores. Secreted to the medium, these bactericidal and bacteriostatic compounds generate a selective advantage for the Bacillaceae family against closely related species. The structure, classification and mode of action of these antimicrobial compounds are described in several excellent reviews [63,64,82,109].

Here, we focused our attention on antimicrobial compounds produced by *Bacillus* spp. identified in honey.

### 7.1. Bacteriocins of Bacillus Species

Within the *Bacillus* groups in honey, *B. subtilis*, *B. amyloliquefaciens*, *B. licheniformis*, *B. thuringiensis* and *B. cereus* produce strain-specific bacteriocins including subtilin, subtilosin, lichenicidin, lichenin, thuricins and cereins [63,64,82,110] (Table 6). They exert their bactericidal activity by the pore formation and membrane permeabilization, leading to the influx/efflux of ions across the membrane, dissipation of membrane potential, leakage of cytoplasmic components and cell death by cell lysis. Despite the presence of the producer strains in honey, most of these bacteriocins have not been yet detected in honey.

One of the frequently encountered bacteriocins of *Bacillus* spp. is subtilin. Subtilin is a simple, pentacyclic lantibiotic structurally and functionally similar to nisin of *Lactococcus lactis*.

Like nisin, subtilin binds to the cell wall precursor, lipid II, and uses it as a docking molecule. Similarly to nisin, subtilin exert a dual antibacterial effect, by the pore formation and by the inhibition of the cell wall synthesis through binding and blocking lipid II-dependent transport of peptidoglycan subunits [64,110,111,112]. These combined mechanisms make subtilin a potent antimicrobial peptide whose bactericidal action occurs rapidly even at nanomolar concentrations. Subtilin targets a broad spectrum of gram-positive bacteria [64,110,111,112]. Its synthesis is regulated by quorum sensing. Subtilin has not been identified in honey thus far.

Some bacteriocins of *Bacillus* spp. are extensively post-translationally modified. Such group of bacteriocins comprises sactipeptides, named that way due to the presence of an unusual thioether bridge, sactionine (Figure 3). This thioether bridge is formed by intramolecular linkage between cysteine sulfur of one amino acid with the α-carbon of another residue [113]. The sactionine seemed to be a crucial structure for antibacterial activity of these bacteriocins [114,115]. The best-known representative of sactipeptides is subtilosin of *Bacillus amyloliquefaciens* and thuricin of *B. thuringiensis*. Although *Bacillus amyloliquefaciens* has been identified in honey microbiota [60,61,62], the subtilosin has not been detected yet in honey.

*B. thuringiensis* strains found in honey produce two sactipeptides, thurincin H and thuricin CD [116]. So far, only thuricin H has been found in honey [116] (Figure 3). The bactericidal mode of action of thuricins is associated with the pore formation. Thuricins shows high levels of bactericidal activity against closely related Bacillus species (with exception of B. cereus), *Geobacillus stearothermophilus*, *L. monocytogenes*, *Listeria innocua*, *Listeria ivanovii*, *S. aureus* and *Carnobacterium maltaromaticum*. Thuricins and other *Bacillus* lantibiotic bacteriocins exhibit narrow-spectrum antimicrobial activity limited only to Gram-positive bacteria. Thuricin CD gained its importance for the efficient killing of *Clostridium difficile* strains [117].

#### Bacteriocins and Autolysins

Bacteriocins of *Bacillus* spp. are indirectly implicated in inducing autolysins and triggering autolysis by the autolysin’s self-digestion of the cell wall. Autolysins are peptidoglycan hydrolases that are naturally involved in the cell-wall turnover and remodelling. During cell growth, they perform the essential autolytic events by selective cleavage of the existing peptidoglycan in order to insert new peptidoglycan subunits into the cell wall [118]. The autolysin-controlled peptidoglycan maturation is not only required for cell shape, cell growth, cell division and separation, but also for motility, chemotaxis and pathogenicity [119]. However, the inhibition of peptidoglycan synthesis by binding of lantibiotic bacteriocins to lipid II leads to an imbalance between synthesis of peptidoglycans and wall autolysis. This imbalance can cause degradation of cell wall, its rupture and eventually cell death. Thus, final consequences are similar between bacteriocins and β-lactam antibiotics actions as they both result from the impairments of peptidoglycan synthesis [120].

Table 6 presents list of *Bacillus* spp. of honey and bacteriocins they could be expected to be produced by these strains.

**Table 6:** Putative antimicrobial compounds produced by *Bacillus* spp. in honey.

Species	Ribosomal Peptides	Nonribosomal Peptides				Target	Ref.
		Antibiotics	Lipopetides	Siderophores	Polyketides		
*B. subtilis*	subtilin						[64,110,111,112]
	subtilosin A					*Gram+* *L.monocytogenes* *Gardnerella vaginalis* *S. agalactiae*	[64,110,111,112]
	sublancin					*B. cereus* *S. pyogenes* *S. aureus*	[64,110,111]
			surfactin			Bacteria, viruses fungi	[64]
			fengycin			fungi	
			bacillomycin			bacteria	[64]
				bacillibactin			[111]
		bacitracin				Gram+ PP synthesis C55-PP carrier	[64,111]
		bacilysin				Gram+, PP synthesis fungi	
					bacillaene		
*B. licheniformis*	lichenin	bacitracin				PP synthesis, Gram+	[110,111]
	lichenicidin					*L. monocytogenes* MRSA VRE	[110]
			lychenisin				[111]
*B. amyloliquefa-ciens*	amylolysin		iturin		bacillaene		
		bacilysin				*S. aureus*	[111]
	Subtiliosin		fengycin				
			surfactin				
*B. cereus*			cereins			*B. cereus*, *B.coagulans*, *B. subtilis*, *B. pumilus*	[110,111]
				bacillibactin			
			thuricin				[110,111]
*B. thuringiensis*			thuricin 17			*B.thuringiensis*, *B. cereus* *E. coli* MM294	[110,111]
			thurincin H			*B. cereus*, *B. subtilis*, *B. megaterium*, *L. monocytogenes*, *L. innocua*, *L. ivanovii*, *S. aureus*, *Carnobacterim psicola*, *Geobacillus stearothermophillus*	[115]
			thuricin CD			*C. difficile*	[117]
*B. mycoides*							
*B. pumilis*	pumilicin		surfactin				
		bacilysin					[110]
			Pumilacidin				[112]
		bacitracin					
*B. safensis*							
*B. altitudinis*							
*B. mojavensis*							
*B. megaterium*	megacin		surfactin				
			fengycin				
			bacillomycins				[112]
*B. aerius*							
*B. altitudinis*							
*P. alvei*							
*P. larvae*			paenibacterin				
*P. polymyxa*	paeniba-cillin					*Bacillus* spp., *C. sporogenes*, *Lactobacillus* spp., *L. lactis*, *Leuconostoc* *mesenteroides*, *Listeria* spp., *Pediococcus cerevisiae*, *S. aureus* *S. agalactiae*	[110]
				bacillibactin			
					bacillaene		[111]
			polymyxin			Gram-positive Gram-negative	[111]
					paenima-crolidin	*S. aureus*	[111]
*B. brevis*		gramicidin					[112]

### 7.2. Non-Ribosomal Peptide Antibiotics of Bacillus *spp.*

Several *Bacillus* strains possess a high genetic capacity to synthesize non-ribosomal peptides (NRP) and polyketide. Up to 8% of genome of some *Bacillus* strains are dedicated to the production of these compounds. Non-ribosomal peptides are synthesized by mutli-complex synthtases and polyketide synthesases as linear, branched or cyclic structures [63,64,82,109,110,111]. The structural and functional diversity of NRP and polyketides targets a range of cellular targets on competing species, giving *Bacillus* strains a significant survival advantage. 

#### 7.2.1. Antibiotics

Among antibiotics, bacitracin, synthesized by *B*. *licheniformis* and *B.subtilis* is a cyclic peptide containing several D-amino acids and a thiazoline ring (Figure 3). The thiazoline ring plays a key functional role in blocking peptidoglycan synthesis by binding to lipid II [121]. Bacitracin has a narrow spectrum activity targeting only gram-positive bacteria including staphylococci, streptococci and *Clostridia*.

Bacilysin is another peptide antibiotic produced by *B. subtilis*, *B. amyloliquefaciens and B. pumilus*. Bacilysin is a small, NRP dipeptide containing in its structure an unusual, non-proteinogenic amino acid L-anticapsin. L-anticapsin is responsible for bacilysin bactericidal action (Figure 3). After uptake of bacilysin by a target susceptible microorganism, L-anticapsin is proteolytically released form dipeptide and serves as a competitive inhibitor of glucosamine synthase which is involved in mannoprotein or peptidoglycan synthesis in fungi and bacteria, respectively. The irreversible inhibition of glucosamine synthase is responsible for inducing the lysis of the microbial cell wall [122]. Bacilysin showed a broad-spectrum antibacterial activity against plant pathogens and against *Candida albicans* [123]. Bacilysin has a broad spectrum of activity against gram-positive and fungal pathogens.

Bacillaene is a small, hybrid non-ribosomal/polyketide antibiotic produced by *Bacillus subtilis* and in *Bacillus amyloliquefaciens* FZB 42 (Figure 3). Bacillaene has an unusual, linear polyene structure consisiting of of six conjugated carbon–carbon double bonds and two amide bonds. Its complex structure is synthesized by three giant polyketide synthases that form an enzymatic complex of the size of ribosomes [124,125].

The bacillaene antibacterial action targets both gram-positive and negative bacteria including human pathogens such as *Serratia marcescens*, *Klebsiella pneumoniae*, *E scherichia coli* and *Staphylococcus aureus.* It displays antifungal activity against *Trichoderma*, *Coriolopsis* and *Fusarium* sp. [126].

#### 7.2.2. Lipopetide Surfactants

Genera of *Bacillus* and *Paenibacillus* are known producers of lipopetides with anti-fungal and antibacterial activities. Lipopeptides are non-ribosomally synthesized cyclic peptides containg β-hydroxy fatty acid chains. This group includes iturin, fengycin, surfactin and bacillomycin (Figure 3). They are abundantly synthesized by *B. amyloliquefaciens*, *B. subtilis*, *B. licheniformis*, *B. pumilus* as well as *Paenibacillus* spp., such as *P. polymyxa*, *P. larvae* [63,64,82,111,127]. Due to amphiphilic structure, lipopeptides act as membrane- active biosurfactants that lower the surface tension of the membrane lipid bilayer. Similarly to biosurfactant of lactobacilli, surfactin, and fengycin prevent the attachment and biofilm-formation by competing species and facilitate the dispersal of established biofilms including human pathogen *Salmonella enterica* [30,64,111,128,129].

Surfactin is recognized as the most active membrane solubilizing compound among lipopetides [130]. Surfactin shows a broad-spectrum of antibacterial activity against plant pathogens such as *Pseudomonas syringae* or *Ralstonia solanacearum* [131]. Surfactin and its metal complex were found to be very effective in dispersal of *C. albicans* biofilm by decreasing cellular surface hydrophobicity [132,133].

Fengycin and iturin display a strong antifungal activity due to the formation of transmembrane channels in fungal membranes leading to cell lysis [134,135].

A new *Bacillus* strain recently isolated from honey, *Bacillus* BH072, has been shown to produce iturin with pore-forming ability [136].

#### 7.2.3. Siderophores

The antimicrobial activity of siderophores is exerted by sequestering iron from the environment, thereby depleting supply of iron to competing microorganisms and inhibiting their growth and proliferation. Since most of microorganisms produce siderophores, the structure of siederophore and its efficiency of iron binding provide a crucial advantage in its antagonistic action against other competitors. Siderophores are synthesized by the large non-ribosomal peptide syntheases complex. The three types of siderophores are classified based on their functional groups, being catecholates, hydroxamates, and α-hydroxy carbolates [137]. Among *Baclillales*, the genera *Bacillus* and *Paenibacillus*, synthesize bacillibactin that contain three catechol groups and therefore efficiently chelate Fe III (ferric iron) [137,138]. Bacillibactin is produced by *B. subtilis*, *B. licheniformis*, *B. anthracis*, *B. cereus* and *B. thuringiensis* under low iron availability for more efficient in iron [139].

Bacillbactin has been isolated from honey bees infected with *P. larvae* [140]. Whether bacillbactin contribute to *P. larvae* pathogenicity has to be investigated. Figure 3 presents the structure of bacitracin, bacilysin, iturin or surfactin, and polyketide bacillaene.

### 7.3. Paenibacillus

The genus *Paenibacillus* is widely distributed in hives of honey bees and nests of wild solitary bees [141]. Several *Paenibacillus* strains are considered pathogenic for bees. *P. alvei* is often found together with *P. apiarius* to accompany *Melissococcus plutonius*, a cause of European foulbrood [127,142]. Another *Paenibacillus* strain, *P. larvae* gained notoriety as a lethal infectious agent of American foulbrood disease [143]. So far, three *Paenibacillus* strains were found in honey, *P*. *alvei*, *P. polymyxa* and *P. larvae* [25,61,144,145].

*Paenibacillus* species produce a structurally diverse group of antimicrobial compounds including lantibiotics bacteriocins such as pediocins and paenibacillin [110,146], non-ribosomal, cyclic lipopeptides-polymyxins and paenibacterin [147] and also putative sactipeptides [148].

Pediocins and paenibacillins are pore-forming lantibiotics displaying broad-spectrum activity against foodborne pathogens [127]. However, polymyxins produced by *P.* *polymyxa* are the best known lipopetides of clinical significance [149]. 

Polymyxins are polycationic, cyclic peptides with a short protruding N-terminus to which a fatty acid chain is covalently attached (Figure 3). The general mechanism of bactericidal action of polymyxins is similar to the pore-forming bacteriocins and include several steps: the binding of hydrophobic fatty acid chain of polymyxin to the lipid A of the lipopolysaccharide of OM of Gram-negative bacteria, destabilization of OM by the interactions with the lipid A, a self-promoted crossing of the OM and the interaction with the cytoplasmic membrane, dissipation of the proton force potential, membrane permeabilization and leakage of the cell content, followed by cell lysis [110]. Thus, in contrast to *Bacillus* lipopeptides, iturin and thuricin, polymyxins target the outer membrane of Gram-negative bacteria. Polymyxins have been used as a last-resort treatment of infections caused by multidrug-resistant Gram-negative bacteria. Among polymyxins, polymyxin B and colistin (polymyxin E) show strong antagonistic activity against Enterobacteriaceae family, including *E. coli*, *Enterobacter* spp., *Klebsiella* spp., *Citrobacter* spp., *Salmonella* spp. and *Shigella* spp. *Acinetobacter baumannii*, *Pseudomonas aeruginosa* and *Stenotrophomonas maltophilia*. They are not active against against Gram-negative cocci (*Neisseria* spp.) or Gram-positive bacteria [150,151].

It has to be noted that polymyxins have been shown to be also synthesized by *P. alvei* [127]. Both *P. polymyxa* and *P. alvei* has been demonstrated to be relevant to honey antimicrobial activity. *P. polymyxa* TH13 isolated from honey produced antimicrobial compound that showed a broad range of antibacterial activity against foodborne pathogens and importantly, against *P. larvae* ssp. ATCC 25747. The antimicrobial compound produced by *P. polymyxa* TH13 was identified as polymyxin E [144].

The presence of *P. alvei* in honey has been reported in several studies but its contribution to honey antibacterial activity was unknown [60,61,127]. However, recently *P. alvei* MP1isolated from buckwheat honey has been shown to exhibit antagonistic activity against reference strains of *L. monocytogenes*, *S. aureus L1—0030* and *E. coli* O157: H7. The isolated antimicrobial protein contains hydrophobic domain resembling a lipopeptide [61] similar to other lipopeptides of *Paenibacillus* strains [149,151]. The efforts are made to elucidate the chemical structure of this lipopeptide [61].

In addition to antibacterial compounds, *Paenibacillus* produces a range of hydrolytic enzymes attacking fungal cell walls such as glucanses, chitinases, cellulases and proteases that are involved in the destruction of cell walls of *Fusasrium* spp. [64,127].

### 7.4. Antimicrobial Compounds of Fungal Origin and Their Potential Contribution to Honey Antimicrobial Activity

Fungi and yeast developed competitive strategies to respond to the challenges from microorganisms occupying the same niche such as the production of secondary metabolites with antimicrobial activities to prevent growth of competitors and exploiting and depleting nutritional resources. Moreover, the end-products of carbohydrate fermentation create antimicrobial environment [69,70,152]. Among the antimicrobial compounds produced by fungi and yeast are mycotoxins, antibiotics, siderophores and surfactants.

#### 7.4.1. Mycotoxins

Mycotoxins (killer toxins) are produced in nectar by yeast including genera *Metschnikowia*, *Zygosaccharomyces*, *Saccharomyces* and *Candida* and filamentous fungi including *Aspergillus*, *Fusarium* and *Penicillium* species. Mycotoxins are always lethal due to inhibition of essential cellular functions such as inhibition of the DNA synthesis, cell wall synthesis or disruption of cell membrane function [70,153]. For instance, zygocin, produced and secreted by the yeast *Zygosaccharomyces bailii* effectlively kills *Candida albicans*, *Candida krusei* and *Candida glabrata* clearing the space for their own growth [154]. While yeast mycotoxins are preferentially directed against filamentous fungi, in contrast, mycotoxins produced by the *Aspergillus*, *Fusarium* and *Penicillium* are of broader spectrum, and can also be deleterious to bees [155], bacteria [156] and plants [157]. Mycotoxin contaminations of food are widespread despite adhering to good agricultural and manufacturing practices in the food chain. Mycotoxins such as aflatoxins are often found in bee product such as pollen, bee larvae, bee bread and whole bees. However, importantly, they are not present at detectable levels in the unprocessed honey. Research showed that lactic acid bacteria and *Bacillus* spp. of honey possess the robust enzymatic system involved in degradation and chemical conversion of mycotoxins to non-toxic derivatives [68,158,159].

#### 7.4.2. β-lactams

The genera of *Aspergillus* spp. and *Penicillium* spp. are known producers of the β-lactam antibiotics (penicillin and cephalosporin) [160] that inhibit the transpeptidase activity of the penicillin binding proteins that cross-linked adjacent peptidoglycan chains during cell wall synthesis. 

Interestingly, in some fermented foods like cheese of cured meat, the growth of *Penicillium* spp.was shown to be associated with the production and secretion of penicillin into the food [161]. Whether or not beta- lactams can be produced by the nectar-dwelling *Penicillium* spp. and carried-over to honey need to be investigated.

#### 7.4.3. Surfactants

Some *Candida* species (*Candida apicola*, *Stramerella bombicola* and *Rhodotorula bogoriensis*) produce sophorolipids that have anti-biofilm activity. Their surfactant properties inhibit the adhesion, biofilm formation and cause dispersion of mature biofilms of *Candida* and *Pichia* species and Gram-positive bacteria [162]. Surfactants of different ecological origins (fungal and bacterial) carried-on to honey might act synergistically, adding to honey’s known anti-biofilm effect [163,164].

#### 7.4.4. Siderophores

Several yeasts secrete siderophores during periods of iron starvation, when the intracellular iron levels decrease to concentrations lower than 10^−6^ M required for microbial growth [165]. In response to iron limitations, yeast and molds similarly to bacteria produce and secrete siderophores containing the catecholate or phenolate groups in their structure, that efficiently chelate iron Fe (III) and other metals like manganese and zinc, from surrounding environment. Iron acquisition system of siderophores and transporters is then one of strategies to prevent growth and multiplications of other, competing microorganisms by depleting this life-supporting metal ion [165].

**Part C**

## 8. The Antagonistic Interactions between Microbes at the Ecological Level

While there is a growing understanding of the contribution of the secondary metabolites of the core honey microbiota to the pool of honey antimicrobial compounds, surprisingly little is known about input of the secondary metabolites generated from the microbe-plant host and microbe-honey bee interactions to this pool. It is plausible that a widespread co-habitation of nectar, bees and honey by yeast and bacteria and their antagonistic interactions might leave their footprint in the form of antimicrobial compounds in honey. The shotgun metagenomics analysis of environmental DNA in honey supported the presence of DNA signatures from plants from which honey originate, DNA of yeast and bacteria residing in honey, and DNA of microbiota of bee alimentary tract, thus portraying honey as the ecological niche that hosts diverse microbial communities [44].

## 9. Pathogenesis-Related Proteins of Plants

The invasion of plant or honey bees by pathogenic microorganisms evokes innate immune responses to counter/resist the attack. The resistance in plants against pathogen is controlled by *R* genes that produce pathogenesis-related proteins and peptides to directly suppress the growth and spread of the pathogen. The PR proteins include peptides such as defensins (PR-12), thionins (PR-13), thaumatin-like (PR-5) and lipid transfer proteins (PR-14) that have broad antibacterial and antifungal activities [166,167]. The primary targets of most PRs are fungal cell wall or bacterial cell envelope. Among carbohydrate-degrading enzymes targeting fungal cell wall are chitinases (PR-2, -4, -8 and -11) and glucanases (PR-2) [166]. PR-8 group of chitinases contains also lysozymes hydrolyzing peptidoglycan (PG) of bacterial cell envelope. PR-10 family possesses ribonuclease activity and might be active against RNA viruses.

A large portion of PR proteins comprise enzymes involved in a breakdown of fungal cell wall as well as enzymes involved in generation of radical oxygen species [14]. The latter includes a nectar redox cycle system that produces biocidal concentrations of hydrogen peroxide [168]. Phenols, flavonoids, terpenes and alkaloids are also included in the group of defence-related compounds. Honey proteomic analysis and the combination of zymography and 2D SDS-PAGE [169,170] showed in honey several proteolytic enzymes originating from nectars, specifically serine-proteases such as trypsin, chymotrypsin and trypsin-and chymotrypsin-like enzymes. Anti-fungal activity of honey might result at least in part from the plant-defence molecules identified in honey.

## 10. Honey Bee Antimicrobial Peptides of Honey

Similarly, in bees, infection with bee pathogens results in cellular and humoral immune defences. Honey bees are prone to invasion by diverse pathogens; bacteria, fungi, viruses and arthorpodes (mites). The common, lethal pathogens of bees are *Paenibacillus larvae* and the fungus *Ascosphaera apis* [171]. Surprisingly, bees’ humoral responses are limited to the production of only a couple of antimicrobial peptides such as proline-rich apidaecins [172] and abaecins [173], cysteine-rich defensins 1 and 2 [174,175] and glycine-rich hymenoptaecin [176].

Until now, antimicrobial peptides of honey bee origin found in honey include defensin-1, hymenoptaecin and jelleins. Klaudiny et al. (2005) [175] detected two variants of defensins genes, *defensin 1* and *defensins 2* in honey bee that were differentially expressed. Their gene products defensin-1 protein has been found in honey [177,178,179] while defensin 2, royalisin, in royal jelly [179]. Despite overlapping sequence and structural features, defensin1 and royalisin present distinct spectrum of antimicrobial activity. While both defensins inhibit growth of Gram-positive bacteria and fungi [180,181], royalisin also inhibits *Paenibacillus larvae larvae* [180,181,182].

Another antimicrobial peptide found in honey is hymenoptaecins. Hymenoptaecins are inducible antimicrobial peptides synthesized after bacterial infection of bees. Initially expressed in the bee fat body and hemocytes, they accumulate rapidly in hemolymph of adult bees and in brood. Similarly to defensins and jelleins, hymenoptaecins require proteolytic processing to be releases in the active form [176,183]. It is believed that similarly to defensins, hymenoptaecins are introduced to honey with the secretion of the bee hypopharyngeal glands. The contribution of this peptide to honey antimicrobial activity has not been directly verified.

Jelleins are small antimicrobial peptides located in the C-terminal portion of the Major Royal Jelly Protein 1, the most abundant protein in honey [184]. They have to be relased proteolytically from the MRJP1 to perform their pore-forming action of the cell membrane of Gram-positive bacteria [184,185] In general, jelleins showed higher antibacterial efficacy against Gram-positive bacteria (*S. aureus*, *B. subtilis*) than against gram-negative bacteria (*E. coli*, *P. aeruginosa*, *K. pneumoniae*). They were also active against fungi, *C. albicans* [185]. Despite their in vitro antibacterial activities against several multi-drug resistant clinical isolates, their contribution to honey antimicrobial activity remains to be established [186]. 

## 11. Conclusions

This review highlights the role of honey microbiota in the production of plethora of antimicrobial compounds. It presents honey as a well-defined habitat with distinct physico-chmical properties that shapes the microbial community structure and richness. The habitat is occupied by microbiota acquired by horizontal transfer from nectar, pollen, bees and environment. The initial diversity of microbial populations is reduced by the change in physicochemical properties during the nectar to honey transformation, which eliminates transient contaminants and less adapted species (such as *Lactobacillus* and *Gluconobacter* genera, and the genera of *Cladosporium*, *Aspergillus*, *Candida*) [4,40,89]. In the end, the core microbiota are comprised of xerotolerant and osmotolerant fungal and bacterial species dominanted by *Bacillus* and *Lactobacillus* spp. and yeasts *Metschnikowia*, *Saccharomyces*, *Zygosaccharomyces* and *Alternaria* spp.

The antagonistic interspecies interactions induced by the overlap of microbial species in honey result in the production and release of diffusible molecules secreted by these species directly to the growth medium, that is, honey. Together, honey microbiota and the products of their metabolic activity comprise honey microbiome. Honey microbiome is a functional unit, in which living microbiota interact with each other and secrete the variety of specialized metabolic compounds including those with antimicrobial properties. 

**Figure 4:**
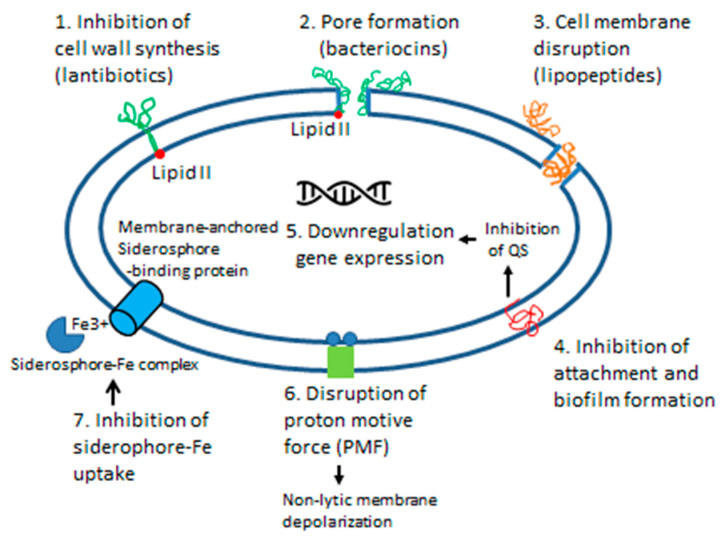
Schematic representation of the effects of antimicrobial compounds (bacteriocins, lipopeptide surfactants and siderophores) produced by honey microbiota on the cytoplasmic membrane integrity and bacterial cell function.

The described antimicrobial compounds produced by the honey core microbiota include antibiotics (β-lactams, bacitracin, bacilysin), antmicrobial peptides of bee origin (defensins, hymenoptaecins), bacteriocins produced by LAB and *Bacillus* spp., biosurfactants and siderophores. Each microorganism detected in honey could potentially produce more than one antimicrobial compound with a different mode of action, targeting different cellular structures (Figure 4). Based on the mode of action, bactericidal effects of antimicrobial peptides, antibiotics or bacteriocins result in the direct membrane damage through the pore-formation, inhibition of peptidoglycan synthesis, and by the increased membrane permeability. The depolarization of the cytoplasmic membrane by biosurfactants (lipopetides, polymixins) and some bacteriocins (bacteriolysins, class III bacteriocins) prevents a multitude of critical cellular function, such as energy production, active transport, signal transduction, quorum sensing, biofilm formation and expression of virulence factors (Figure 4).

Thus, honey microbiome can be considered an ecological reservoir of antmicrobial compounds that are produced and secreted by honey microbiota. In a nutshell, these antimicrobial compounds might include:

1. Cell wall damaging compounds:
  - Bacteriocins originating from *Bacillus* and *Lactobacillus* species
  - Antimicrobial peptides originated from bee: defensins, hymenoptaecins, jelleins
  - Antimicrobial peptides originating from plants: thionins and thaumatin-like peptides
  - Antibiotics; bacilysin and bacillaene of bacterial origin
  - Biosurfactants; lipopetides of bacterial origin (surfactin, iturin, fengycin, polymyxins)
  - Biosurfactants of plant origin; lipid transfer proteins
  - Biosurfactants of fungal origin
  - Anti-fungal enzymes of plant origin: chitinases, glucanases and lysozymes hydrolyzing peptidoglycan (PG) of bacterial cell envelope
2. Inhibitors of peptidoglycan synthesis:
  I. Antibiotics; β-lactams of fungal origin and bacitracin of bacterial origin
  J. Lantibiotic bacteriocins of bacterial origin
3. Siderophores of bacterial and fungal origin

The efforts toward the identification of these antibacterials in honey could bring desirable therapeutic and nutritional outcomes. The caveate is that honey processing technologies and the inactivation of microorganisms by pasteurization kill inadvertently the harmful foodborne pathogens together with the potentially beneficial, probiotic species of *Bacillus*, *Lactobacillus*, *Bifidobacteria* or *Saccharomyces*. It could be argued, however, that the antimicrobial compounds accumulated in honey before sterilization might remain functional anyway. One possible mechanism for their preservation in a native and functional form is by their sequestration into colloidal particles that are formed in honey under low water activity, high sugar concentration and high concentration of macromolecules [187]. The stable, metabolically inactive colloidal assemblies can transiently sequester, stabilize and store bioactive molecules such as enzymes [187] and release them in active form upon honey dilutions. The potential practicality of the colloidal system is its reversibility that ensures “delivery” of sequestered, bioactive molecules when needed [187].

In sum, honey microbiome comprises a new source of putative honey antimicrobial compounds that might constitute the long- sought “unknown”, non-peroxide factors responsible for the residual honey antibacterial activity after removal/blocking of main biocidal activities of H_2_O_2_ or MGO. Thus, honey microbiome is a promising source to discover new antimicrobials.

## Figures and Tables

**Table 1:** Overview of core taxa in honey.

Phylum	Class	Order	Family	Genus
Proteobacteria	Alphaproteobacteria	Rhodospirillales	Acetobacteraceae	*Gluconobacter*
	Gammaproteobacteria	Pseudomonadales	Psedomonadaceae	*Pseudomonas*
		Enerobacteriales	Enetrobacteriacea	*Enterobacter*
				*Escherichia*
				*Klebsiella*
Actinobacteria		Actinomycetales	Micrococcaceae	*Micrococcus*
		Bifidobacteriales	Bifidobacteriaceae	*Bifidobacterium*
			Microbacteriaceae	*Microbacterium*
Firmicutes	Bacilli	Bacillales	Bacillaceae	*Bacillus*
			Paenibacillaceae	*Paenibacillus*
			Staphylococcacea	*Staphylococcus*
		Lactobacillales	Lactobacillaceae	*Lactobacillus*
			Leuconostocacea	*Fructobacillus*
				*Leuconstoc*
				*Oenococcus*
				*Weisella*
			Streptococcaceae	*Lactococcus*
				*Streptococcus*
			Enterococcacea	*Melissococcus*
		Clostridiales	Clostridiaceae	*Clostridium*

Table 1 is based on data from studies of Anderson et al., 2013 [42], Corby-Harris et al., 2014 [43], Bovo et al., 2018 [44] and Manirajan et al., 2016 [45].

**Table 2:** Members of Lactobacillales identified in honey bee foregut (crop) and honey.

Order	Family	Genus	Species
Lactobacillales	Lactobacillaceae	*Lactobacillus*	*L. acidophilus*
			*L. apis*
			*L. apinorum*
			*L.jensenii*
			*L. brevis*
			*L. florum*
			*L. helsingborgensis*
			*L. johnsonii*
			*L. kefiranofaciens*
			*L. kimbladii*
			*L. kullabergensis*
			*L. mellifer*
			*L. mellis*
			*L. melliventris*
			*L. kunkeei*
			*L. plantarum*
			*L. rossiae*
			*L. versmoldensis*
	Leuconostocaceae	*Fructobacillus*	*F. fructosus*
		*Leuconostoc*	
		*Oenococcus*	
		*Weissella*	
	Pediococcaceae	*Pediococcus*	
	Streptococcaceae	*Streptococcus*	
	Enterococcaceae	*Enterococcus*	*E. faecalis*
			*E.faecium*
		*Aerococcus*	
		*Carnobacterium*	

Table 2 is based on the results from Endo et al., 2009 [46], Endo, Salminen, 2013 [47], Neveling et al., 2012 [48], Olofsson, Vásquez, 2008 [49] and Olofsson et al., 2014 [50].

**Table 3:** Composition of the family Bacillaceae in honey identified by genotyping.

Family	Genus	Species
Bacillaceae	*Bacillus*	*B. subtilis*
		*B.* *methylotrophicus*
		*B. atrophaeus*
		*B. licheniformis*
		*B. amyloliquefaciens*
		*B. cereus*
		*B. thuringiensis*
		*B. mycoides*
		*B. pseudomycoides*
		*B. weihenstephanen*
		*B. pumilis*
		*B. safensis*
		*B. altitudinis*
		*B. mojavensis*
		*B. anthracis*
		*B. aerius*
		*B. xiamenensis*
		*B. wiedmannii*
		*B. proteolyticus*
		*B. tropicus*
		*B. circulans*
		*B. flexus*
		*B. zhangzhouensis*
	*Lysinibacillus*	*L. fusiformis*
		*L. macroides*
		*L. pakistanensis*
		*L. boronitolerans*
	*Oceanobacillus*	
Paenibacillaceae	*Paenibacillus*	*P. alvei*
		*P. larvae*
		*P. polymyxa*
		*P. apiarius*
	*Brevibacillus*	*B. brevis*
		*B. limnophilus*
Listeriaceae		*L. monocytogenes*
Staphylococcaceae		*S. epidermidis*
		*S. caprae*
		*S. pasteuri*

Table 3 is based on results obtained by Pomastowski et al., 2019 [61], Sinacori et al., 2014 [59], Pajor et al., 2018 [60], Brudzynski, Flick, 2019 [62].

**Table 4:** Fungi and yeasts found in honey.

Division	Class	Oder	Family	Genus	Species
Ascomycota	Eurotiomycetes	Eurotiales	Trichocomaceae	*Aspergillus*	*A. pseudoglaucus*
					*A. asperescens*
					*A. montevidensis*
					*A. flavus*
					*A. versicolor*
					*A. niger*
					*A. fumigatus*
				*Penicillium*	*P. camemberti*
					*P. citrinum*
					*P. corylophilum*
					*P. cravenianum*
					*P. apimei*
				*Talaromyces*	
			Monascaceae	*Monascus*	*M. pilosus*
					*M. mellicola*
					*M. purpureus*
					*M. ruber*
		Ascosphaerales	Ascosphaeracea	*Bettsia*	*B. alvei*
					*Ascosphaera apis*
		Onygenales	Myxotrichaceae	*Skoua*	*Skoua fertilis*
				*Oidiodendron*	
			Eremascaceae	*Eremascus*	*Ermascus albus*
			Ascosphaeriacea	*Ascosphaera*	*Ascosphaera atra*
					*Ascosphaera apis*
			Spiromastigaceae		
		Schizosaccharomy-cetales	Schizosaccharomyce-taceae	*Schizosaccharomyces*	*S. octosporus*
	Saccharomycetes	Saccharomycetales	Saccharomycetaceae	*Zygosaccharomyces*	*Z. favi*
					*Z. mellis*
					*Z. richteri*
					*Z. rouxii*
					*Z. siamensis*
				*Candida*	*C. lundiana*
					*C. magnoliae*
					*C. sorbosivorans*
					*C. suthepensis*
				*Saccharomyces*	*S. cerevisiae*
				*Cyberlindnera*	*C. jadinii (Torula)*
				*Starmerella*	
			Metschnikowiaceae	*Metschnikowia*	
	Dothideomycetes	Capnodiales	Davidiellaceae	*Cladoisporium*	
		Pleosporales	Pleosporaceae	*Alternaria*	*A. multiformis*
				*Stemphylium*	
	Sordariomycetes	Hypocreales	Nectriaceae	*Fusarium*	
Mucoromy-cota		Mucorales		*Mucor*	*M. ruber*
					*M. plumbeus*

Table 4 is based on the results of Rodríguez-Andrade, et al., 2019 [67], Kačániová et al., 2012 [68] and Sinacori et al., 2014 [59].

**Table 5:** Putative bacteriocins produced by lactobacilli detected in honey and honey bee.

Species	Bacteriocins	Target	Ref.
*L. acidophilus*	acidocin	*Lactobacillus* sp. *Listeria monocytogenes* *Enterococcus faecalis*	[85,90]
	lactacins	*Lactobacillus fermentum* *Enterococcus faecalis* *Lactobacillus delbrueckii* *Lactobacillus helveticus* *Lactobacillus debrweckii* *Lactobacillus helveticus* *Lactobacillus.bulgaricus.* *Lactococcus lactis.*	[90]
*L. helveticus*	helveticin J	*Lactobacillus* *Lactobacillus bulgaricus* *Lactococcus lactis*	[85,91]
	lactocin 27		[90]
*L. johnsonii*	lactacin F		[77]
*L. kunkeei*	kunkicin		
*L. plantarum*	plantaricin	*Listeria monocytogenes Staphylococcus* *aureus*, *Salmonella typhimurium* and *Escherichia coli*	[92]
		*Bacillus cereus*, *B. pumilus*, *B. megaterium*, *Pediococcus*, *Carnobacteria*, *Clostiridia* and *Propionobacteria*	
*L. lactis*	nisin	*Staphylococcus aureus*, *Listeria innocua*, *Lactobacillus sakei*, *Lactobacillus plantarum*, *Bacillus* spp. *Micrococcus* spp. *Clostridium* spp.	[85,93]
	lacticin 3147	*Clostridium* sp. *Listeria monocytogenes* *Staphylococcus aureus* MRSA VRE *Enterococcus faecalis* *Propionibacterium acne* *Streptococcus mutans*	[85]
*Pedicoccus pentosaceus*	pediocin	*Listeria monocytogenes* *Lactobacillus* *Lactococcus* *Leuconostoc* *Pediococcus* *Staphylococcus* *Enterococcus* *Listeria* *Clostridium*	[63,85]
